# Nitric oxide stress and activation of AMP-activated protein kinase impair *β-*cell sarcoendoplasmic reticulum calcium ATPase 2b activity and protein stability

**DOI:** 10.1038/cddis.2015.154

**Published:** 2015-06-18

**Authors:** X Tong, T Kono, C Evans-Molina

**Affiliations:** 1 Department of Cellular and Integrative Physiology, Indiana University School of Medicine, Indianapolis, IN 46202, USA; 2 Department of Medicine, Indiana University School of Medicine, Indianapolis, IN 46202, USA; 3 Department of Biochemistry and Molecular Biology, Indiana University School of Medicine, Indianapolis, IN 46202, USA; 4 The Herman B Wells Center for Pediatric Research, Indiana University School of Medicine, Indianapolis, IN 46202, USA; 5 The Roudebush VA Medical Center, Indianapolis, IN 46202, USA

## Abstract

The sarcoendoplasmic reticulum Ca^2+^ ATPase 2b (SERCA2b) pump maintains a steep Ca^2+^ concentration gradient between the cytosol and ER lumen in the pancreatic *β-*cell, and the integrity of this gradient has a central role in regulated insulin production and secretion, maintenance of ER function and *β*-cell survival. We have previously demonstrated loss of *β-*cell SERCA2b expression under diabetic conditions. To define the mechanisms underlying this, INS-1 cells and rat islets were treated with the proinflammatory cytokine interleukin-1*β* (IL-1*β*) combined with or without cycloheximide or actinomycin D. IL-1*β* treatment led to increased inducible nitric oxide synthase (*iNOS*) gene and protein expression, which occurred concurrently with the activation of AMP-activated protein kinase (AMPK). IL-1*β* led to decreased SERCA2b mRNA and protein expression, whereas time-course experiments revealed a reduction in protein half-life with no change in mRNA stability. Moreover, SERCA2b protein but not mRNA levels were rescued by treatment with the NOS inhibitor l-NMMA (*NG*-monomethyl l-arginine), whereas the NO donor SNAP (*S*-nitroso-*N*-acetyl-d,l-penicillamine) and the AMPK activator AICAR (5-aminoimidazole-4-carboxamide ribonucleotide) recapitulated the effects of IL-1*β* on SERCA2b protein stability. Similarly, IL-1*β*-induced reductions in SERCA2b expression were rescued by pharmacological inhibition of AMPK with compound C or by transduction of a dominant-negative form of AMPK, whereas *β-*cell death was prevented in parallel. Finally, to determine a functional relationship between NO and AMPK signaling and SERCA2b activity, fura-2/AM (fura-2-acetoxymethylester) Ca^2+^ imaging experiments were performed in INS-1 cells. Consistent with observed changes in SERCA2b expression, IL-1*β*, SNAP and AICAR increased cytosolic Ca^2+^ and decreased ER Ca^2+^ levels, suggesting congruent modulation of SERCA activity under these conditions. In aggregate, these results show that SERCA2b protein stability is decreased under inflammatory conditions through NO- and AMPK-dependent pathways and provide novel insight into pathways leading to altered *β-*cell calcium homeostasis and reduced *β-*cell survival in diabetes.

The pancreatic *β-*cell requires the maintenance of a robust intraluminal endoplasmic reticulum Ca^2+^ pool to support high levels of protein translation and proinsulin processing. Calcium release from ER stores is also responsible for the activation or augmentation of a number of key *β*-cell signaling pathways, including glucose and incretin-induced insulin secretion.^1^Although release of ER Ca^2+^ occurs through the inositol 1,4,5-trisphosphate and ryanodine receptors (RyRs), intraluminal ER Ca^2+^ levels are largely maintained through activity of the sarcoendoplasmic reticulum calcium ATPase (SERCA) pumps that transport two Ca^2+^ ions into the ER at the expense of one ATP.^2, 3^ In mammals, three different SERCA genes (*ATP2A1*, *ATP2A2* and *ATP2A3*) encode the proteins SERCA1, 2 and 3, and to date, at least 14 isoforms have been identified.^4^ SERCA2b is the most prevalent isoform expressed in the mouse pancreatic islet,^5^ and we and others have demonstrated diminished *β*-cell SERCA2b expression in human and rodent models of type 1 and type 2 diabetes, resulting in Ca^2+^ dyshomeostasis, impaired insulin secretion, activation of ER stress signaling pathways and impaired *β*-cell survival.^5, 6, 7, 8, 9^

Loss of *β*-cell SERCA2b expression under proinflammatory and diabetic conditions is thought to occur, at least partially, through nitric oxide (NO)-dependent mechanisms.^6^ However, whether NO-mediated loss of SERCA2b expression and activity is primarily due to transcriptional or posttranslational mechanisms is not clear. Moreover, the downstream pathways that synergize with NO signaling to alter endoplasmic reticulum Ca^2+^ have not been fully defined. In this regard, the activity and fidelity of ATPases including SERCA are highly dependent on overall cellular energy status. SERCA pumps are estimated to consume 7–25% of total cellular ATP, whereas ADP levels are closely correlated with SERCA activity.^10, 11, 12^ A well-recognized downstream effect of proinflammatory cytokine signaling and NO production is an impairment of mitochondrial function, leading to altered ATP synthesis.^13^ Although these effects contribute to *β-*cell dysfunction and apoptosis, they also lead to the activation of AMP-activated protein kinase (AMPK), which serves as the master sensor of cellular energy status.^14^ AMPK has a wide number of downstream substrates involved in a variety of processes including metabolism, inflammation and ion transport.^15^ Interestingly, AMPK has been shown to regulate directly the activity, expression and cellular localization of other high-energy-consuming protein pumps including the plasma membrane Na^+^,K^+^-ATPase in lung epithelial cells and the H^+^ATPase of the intercalated cells of the kidney.^16, 17, 18^ This level of interaction represents one mechanism through which AMPK signaling may act to limit energy expenditure under stress conditions. Interestingly, a recent proteomics study performed in *β*-cells suggested a direct physical interaction between AMPK and SERCA2,^19^ but a functional relationship between these proteins in the *β-*cell remains unexplored.

Here, we first aimed to define the mechanisms through which NO signaling impacts SERCA2b expression in the pancreatic *β-*cell. Our second goal was to identify the pathways that synergize with NO to regulate *β-*cell ER Ca^2+^ homeostasis under inflammatory conditions, hypothesizing a novel role for chronic AMPK activation in the regulation of SERCA2b activity.

## Results

### The proinflammatory cytokine IL-1*β* decreases SERCA2b protein but not mRNA stability in both INS-1 cells and isolated rat islets

**Figure 1:**
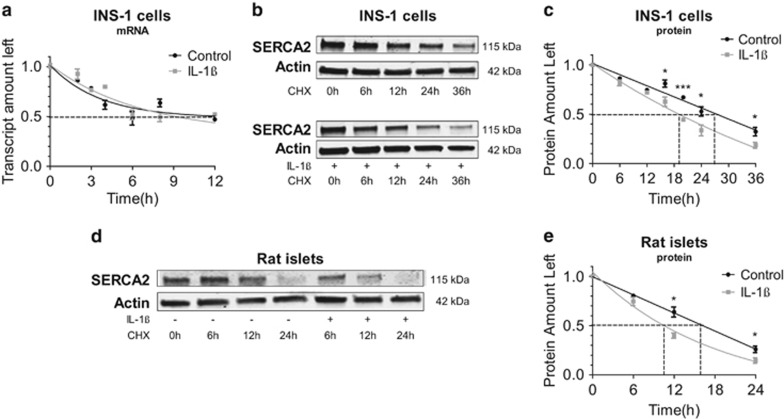
IL-1*β* treatment decreases SERCA2b protein stability in INS-1 cells and isolated rat islets. INS-1 cells (**a**–**c**) or isolated rat islets (**d**–**e**) were treated with 1 *μ*M actinomycin or 10 *μ*M CHX combined with or without 5 ng/ml of IL-1*β* for indicated times. Total RNA and protein were isolated, and RNA was subjected to quantitative real-time PCR (qRT-PCR) for quantification of SERCA2b and actin transcript levels. Immunoblot was performed using antibodies against SERCA2 and actin. Protein and mRNA levels were plotted relative to levels at time zero, and one-phase decay lines for each treatment are shown. Indicated comparisons are significantly different (*P<0.05 and ***P<0.001)

Previous work by our group and others have demonstrated significant downregulation of SERCA2b mRNA and protein levels under diabetic conditions.^5, 6, 7, 8^ To determine whether this was secondary to alterations in either mRNA or protein stability, actinomycin and cycloheximide (CHX) time-course experiments were performed under basal conditions and then following treatment with the proinflammatory cytokine interleukin-1*β* (IL-1*β*). Under control conditions, *β-*cell SERCA2b mRNA exhibited a half-life of ~9 h, and IL-1*β* treatment had no effect mRNA stability (Figure 1a). In contrast, SERCA2 protein in INS-1 cells exhibited a half-life of ~24 h under basal conditions (Figures 1b and c), and IL-1*β* significantly reduced the half-life to ~19 h (Figures 1b and c). In rat islets, the protein half-life was noted to be ~17 h under control conditions, whereas treatment with IL-1*β* significantly reduced the half-life to ~11 h (Figures 1d and e).

### NO-dependent downregulation of SERCA2 occurs at the translational level

**Figure 2:**
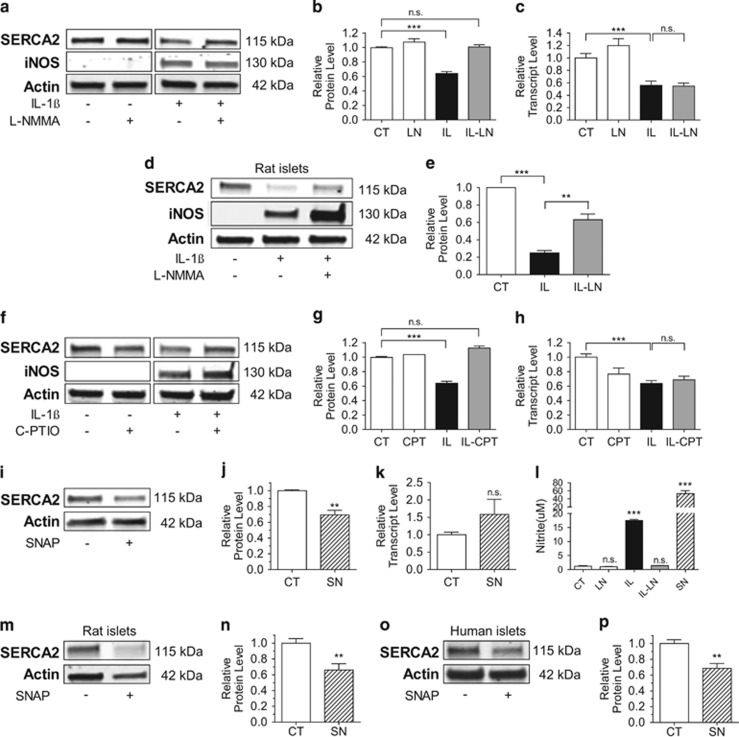
NO-dependent loss of SERCA2 expression occurs at a translational level. INS-1 cells (**a**–**c** and **f**–**h**) or isolated rat islets (**d**–**e**) were treated with dimethyl sulfoxide (DMSO) (CT) or IL-1*β* (IL) combined with or without 0.5 mM of the NOS inhibitor l-NMMA (LN) or 100 *μ*M of the NO scavenger C-PTIO (CPT) for 24 h. Total protein was isolated and immunoblot was performed using antibodies against SERCA2, iNOS and actin. Quantitative protein levels of SERCA2 are shown graphically (**b**, **e** and **g**). Total mRNA was isolated from the INS-1 cells treated with CT, LN, IL, IL-LN or IL-CPT, and reverse-transcribed RNA was subjected to quantitative real-time PCR (qRT-PCR) for quantification of SERCA2b and actin transcript levels (**c** and **h**). Next, INS-1 cells, isolated rat islets or human islets were treated with or without the NO donor SNAP (SN) at 300 mM for 24 h (**i**–**k** and **m**–**p**). Total protein and mRNA were isolated. Immunoblot was performed using antibodies against SERCA2 and actin, and quantitative protein levels are shown graphically (**j**, **n** and **p**). Reverse-transcribed RNA was subjected to qRT-PCR for quantification of SERCA2b and actin transcript levels (**k**). INS-1 culture media was collected at treatment end, and nitrite concentration was measured (**l**). Results are statistically different from control conditions (**j**, **l**, **n** and **p**), or indicated comparisons are significantly different (**P<0.01 and ***P<0.001)

Altered SERCA2b expression under proinflammatory conditions is thought to occur at least partly through NO-mediated signaling pathways.^6^ However, the precise mechanisms underlying NO-mediated alterations in SERCA2b expression have not been fully defined. To this end, INS-1 cells were treated with IL-1*β*, combined with or without the NO synthase (NOS) inhibitor, l-NMMA (*NG*-monomethyl l-arginine), for 24 h.^9^ Following IL-1*β* treatment, loss of both SERCA2b protein and mRNA expression was observed (Figures 2a–c). l-NMMA treatment was able to rescue SERCA2b protein levels (Figures 2a and b). However, no effect was observed on mRNA expression (Figure 2c). These results were confirmed in rat islets (Figures 2d and e), where l-NMMA also resulted in a partial rescue of SERCA2 expression following treatment with the proinflammatory cytokine IL-1*β*.

INS-1 cells were treated next with IL-1*β*, combined with or without the specific NO scavenger C-PTIO (carboxylated derivative of 2-phenyl-4,4,5,5-tetramethylimidazoline-1-oxyl-3-oxide; Figures 2f–h).^10^ Similar to effects observed with l-NMMA, C-PTIO rescued SERCA2 protein but not transcript levels, suggesting IL-1*β*-mediated effects on protein expression were indeed NO-dependent. To define whether NO was then sufficient to exert this effect, INS-1 cells were treated with the NO donor, SNAP (*S*-nitroso-*N*-acetyl-d,l-penicillamine). As expected, SNAP decreased SERCA2 protein to a level almost equivalent to that observed with IL-1*β*, whereas mRNA levels were not significantly altered (Figures 2i–k). Nitrite levels in the culture media were measured under each experimental condition and found to increase with IL-1*β* and SNAP treatment, whereas l-NMMA exhibited the expected effect of decreased nitrite production following IL-1*β* treatment (Figure 2l). Finally, to confirm these results in primary cells, rat and cadaveric human islets were treated with SNAP. Consistent with results observed in INS-1 cells, SERCA2 protein expression was significantly decreased compared with control conditions in both rat and human islets (Figures 2m–p).

### Activation of AMPK*α* Th173 contributes to SERCA2 downregulation at the translational level

**Figure 3:**
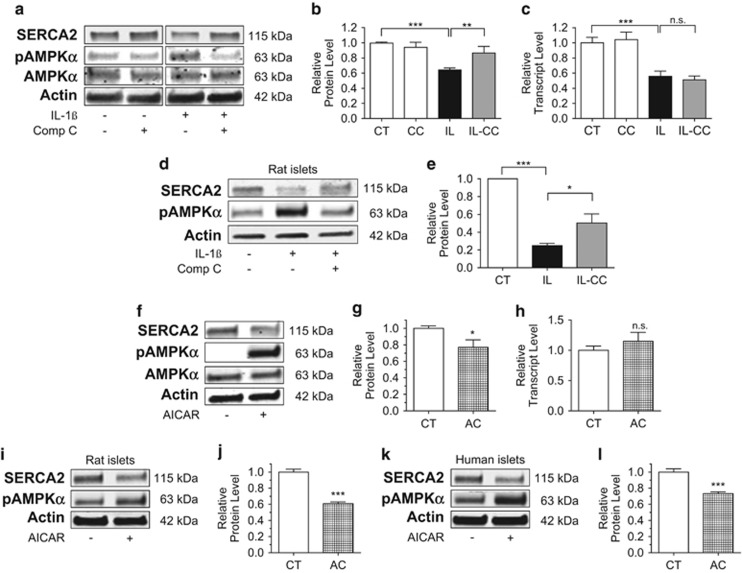
Activation of AMPK*α* Th173 leads to a loss of SERCA2 protein expression. INS-1 cells (**a**–**c**) or isolated rat islets (**d**–**e**) were treated with dimethyl sulfoxide (DMSO) (CT) or 5 ng/ml IL-1*β* (IL) combined with or without 10 *μ*M of the AMPK inhibitor CC for 24 h. Total protein was isolated, and immunoblot was performed using antibodies against SERCA2, phosphorylated AMPK*α* Th173 (pAMPK*α*), total AMPK and actin. Quantitative SERCA2 protein levels are shown graphically (**b** and **e**). Total mRNA was isolated from INS-1 cells treated with CT, IL and IL-CC, and reverse-transcribed RNA was subjected to quantitative real-time PCR (qRT-PCR) for quantification of SERCA2b and actin transcript levels (**c**). Next, INS-1 cells or isolated rat islets and human islets were treated with and without the AMPK activator AICAR (AC) at 2 mM for 24 h (**f**–**l**). Total protein and mRNA were isolated; immunoblot was performed using antibodies against SERCA2, pAMPK*α*, AMPK and actin. Quantitative protein levels of SERCA2 are shown graphically (**g**, **j** and **l**). Reverse-transcribed RNA was subjected to qRT-PCR for quantification of SERCA2b and actin transcript levels (**h**). Indicated comparisons are significantly different (*P<0.05, **P<0.01 and ***P<0.001), or results are statistically different from control conditions (**g**, **j** and **l**)

The primary goal of our study was to next define novel downstream pathways that synergized with NO to influence SERCA2b expression and the overall regulation of ER Ca^2+^ homeostasis. To test whether IL-1*β*-induced SERCA2 downregulation could be linked to an AMPK-mediated effect, INS-1 cells and isolated rat islets were treated with IL-1*β* combined with or without the AMPK inhibitor, compound C (CC). Increased levels of phosphorylated AMPK*α* Th173 were observed following treatment with IL-1*β*, confirming previous findings that proinflammatory cytokines lead to the activation of AMPK signaling.^14^ Similarly, AMPK activation was blocked by CC (Figure 3a). Interestingly, CC was also capable of fully reversing IL-1*β*-mediated loss of SERCA2 protein (Figures 3a and b). Again, these effects appeared to be primarily restricted to protein expression as no significant change in transcript levels were observed (Figure 3c). To confirm this relationship, rat islets were treated with IL-1*β* and CC. Similar to results obtained in INS-1 cells, altered SERCA2 protein expression under inflammatory conditions was prevented by CC (Figures 3d and e). Next, to study whether direct activation of AMPK was sufficient to decrease SERCA2 expression, INS-1 cells were treated with the AMPK agonist AICAR (5-aminoimidazole-4-carboxamide ribonucleotide) for 24 h. Results demonstrated that AICAR indeed decreased SERCA2 protein expression to a level similar to that observed with IL-1*β* and SNAP treatment (Figures 3f and g). Consistent with previous results observed with SNAP, mRNA levels were again unaffected (Figure 3h). Decreased SERCA2 protein expression with AICAR-mediated AMPK activation was confirmed in isolated rat islets and cadaveric human islets (Figures 3i–l). In aggregate, these results indicate that *β*-cell SERCA2 protein half-life is significantly altered under inflammatory conditions and loss of protein expression occurs concomitantly through NO and AMPK-dependent mechanisms.

### NO and activation of AMPK decrease SERCA2 protein stability

**Figure 4:**
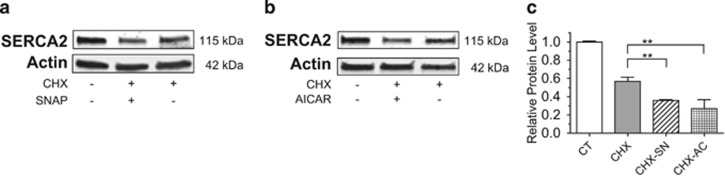
SERCA2 protein stability is decreased by NO-dependent signaling and AMPK activation. INS-1 cells were treated with dimethyl sulfoxide (DMSO) (CT) or 10*μ*M CHX combined with or without 300 mM of SNAP (SN) or 2 mM AICAR (AC) for 24 h. Total protein was isolated; immunoblot was performed using antibodies against SERCA2 and actin (**a** and **b**). (**c**) Quantitative protein levels of SERCA2 are shown graphically. Indicated comparisons are significantly different (**P<0.01)

To address next whether NO- and AMPK-mediated loss of SERCA2 expression specifically resulted from alterations in protein stability, INS-1 cells were treated with CHX combined with or without SNAP or AICAR. Results showed that treatment with both SNAP and AICAR significantly reduced SERCA2 protein half-life (Figures 4a–c).

### AMPK activation is required for IL-1*β*-induced downregulation of SERCA2 protein

**Figure 5:**
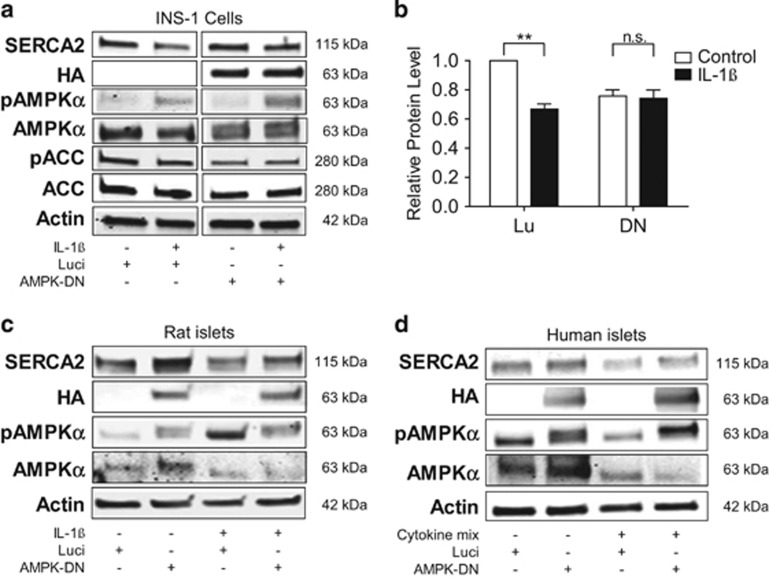
AMPK activation is required for IL-1*β*-induced loss of SERCA2 protein expression. INS-1 cells (**a** and **b**), isolated rat islets (**c**) or non-diabetic cadaveric human islets (**d**) were transduced with an HA-tagged AMPK-DN or DN or control adenovirus (Luci or Lu) before 24- h treatment with or without 5 ng/ml IL-1*β* (for INS-1 cells and rat islets) or a combination of 5 ng/ml IL-1*β*, 1 ng/ml TNF*α* and 100 ng/ml IFN*-γ* in human islets. Total protein was isolated, and immunoblot was performed using antibodies against SERCA2, pAMPK*α*, total AMPK*α*, phosphorylated ACC (pACC), total ACC, HA and actin. (**b**) Quantitative protein levels of SERCA2 in INS-1 cells are shown graphically. Indicated comparisons are significantly different (**P<0.01)

INS-1 cells, rat islets and human islets were next transduced with an HA-tagged AMPK-DN or luciferase expressing control adenovirus to further test the relationship between AMPK signaling and SERCA2 expression. Reduced AMPK signaling with the AMPK-DN construct was confirmed by reduction in the level of phosphorylated acetyl-CoA carboxylase, a key downstream target of AMPK (Figures 5a, c and d).^15^ Virally transduced INS-1 cells and rat islets were treated with IL-1*β*, whereas human islets were treated with a combination of IL-1*β*, IFN-*γ* and TNF-*α* (tumor necrosis factor-*α*). Interestingly, the AMPK-DN adenovirus increased basal expression of SERCA2 in rat and human islets (Figures 5c and d). Moreover, in AMPK-DN-transduced INS-1 cells (Figures 5a and b), rat islets (Figure 5c) and human islets (Figure 5d), cytokine-induced reductions in SERCA2 protein expression were prevented. Taken together, these data demonstrate that AMPK is necessary for proinflammatory-induced downregulation of SERCA2 protein expression.

### AMPK activation modulates IL-1*β-*induced iNOS expression and it is required in SNAP-induced downregulation of SERCA2

**Figure 6:**
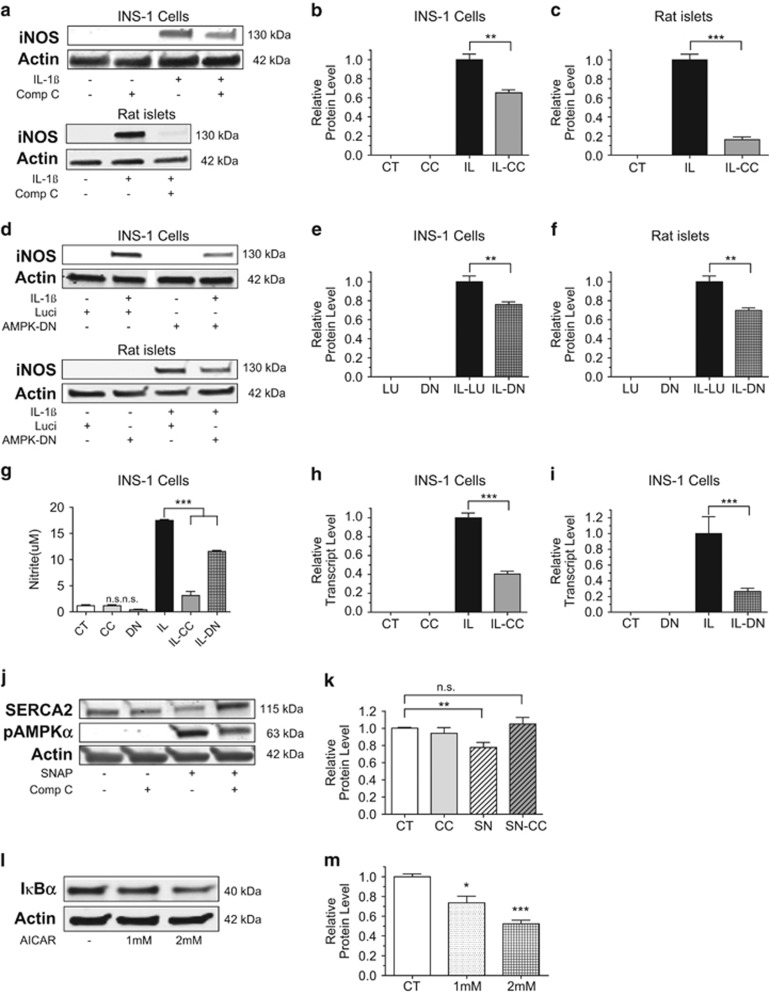
AMPK activation modulates IL-1*β-*induced iNOS expression and is required for SNAP-induced downregulation of SERCA2. (**a**–**i**) INS-1 cells or isolated rat islets were treated with dimethyl sulfoxide (DMSO) (CT), CC, 5 ng/ml IL-1*β* (IL) with or without CC (IL-CC) or transduced with an HA-tagged AMPK-DN or DN or control adenovirus (Luci or Lu) before treatment with or without 5 ng/ml IL-1*β*. Total protein was isolated, and immunoblot was performed using antibodies against iNOS and actin. Quantitative protein levels of iNOS are shown graphically (**b**, **c** and **e**, **f**). Total mRNA was isolated from INS-1 cells, and reverse-transcribed RNA was subjected to real-time PCR for quantification of *iNOS* and *actin* transcript levels (h and **i**). (**g**) INS-1 culture media was collected at treatment end, and nitrite concentration measurement was performed. (**j**–**m**) Next, INS-1 cells were treated with DMSO (CT), 300 mM of SNAP (SN) combined with or without 10 *μ*M of CC, or AICAR at the indicated doses for 24 h. Total protein was isolated, and immunoblot was performed using antibodies against SERCA2, pAMPKa, IκBα and actin. (**k** and **m**) Quantitative protein levels of SERCA2 are shown graphically. Indicated comparisons are significantly different (*P<0.05, **P<0.01 and ***P<0.001)

To study further the relationship between AMPK and NO signaling following cytokine stress, INS-1 cell and rat islets were treated with CC concurrently with IL-1*β*. Interestingly, inducible nitric oxide synthase (iNOS) protein expression was significantly decreased in the presence of CC (Figures 6a–c). Similarly, decreased *iNOS* gene expression was observed in INS-1 cells treated with CC and IL-1*β* (Figure 6h). To rule out nonspecific effects from the use of pharmacological inhibitors, INS-1 cells and rat islets were again transduced with the AMPK-DN or control adenovirus and subsequently treated with IL-1*β*. In AMPK-DN-transduced INS-1 cells and rat islets, a similar reduction in iNOS expression was noted following IL-1*β* treatment, and reduced *iNOS* gene expression was observed (Figures 6d–f, i). This relationship was confirmed by measuring nitrite production, where results showed that IL-1*β*-mediated increases in nitrite production were markedly reduced with CC and partially reduced in INS-1 cells transduced with the AMPK-DN adenovirus (Figure 6g). To define whether AMPK activation was then necessary for SNAP-induced loss of SERCA2 expression, INS-1 cells were treated with SNAP combined with or without CC. Interestingly, SERCA2 protein expression was indeed preserved by CC treatment (Figures 6j and k).

In aggregate, our data showed that AMPK is activated under inflammatory conditions and IL-1*β*-mediated reductions in SERCA2 expression were partially AMPK-dependent. However, our results also showed that AMPK signaling led to an amplification of NO-mediated inflammatory responses in the pancreatic *β*-cell. To investigate this further, INS-1 cells were treated with 1 and 2 mM of AICAR for 24 h. Results showed that AICAR decreased I*κ*B*α* protein levels in a dose-dependent manner, suggesting that AMPK activation leads to reduced tethering of NF*-κ*B in the cytosol (Figures 6l and m).

### AMPK activation alters *β*-cell calcium homeostasis and SERCA2 activity

**Figure 7:**
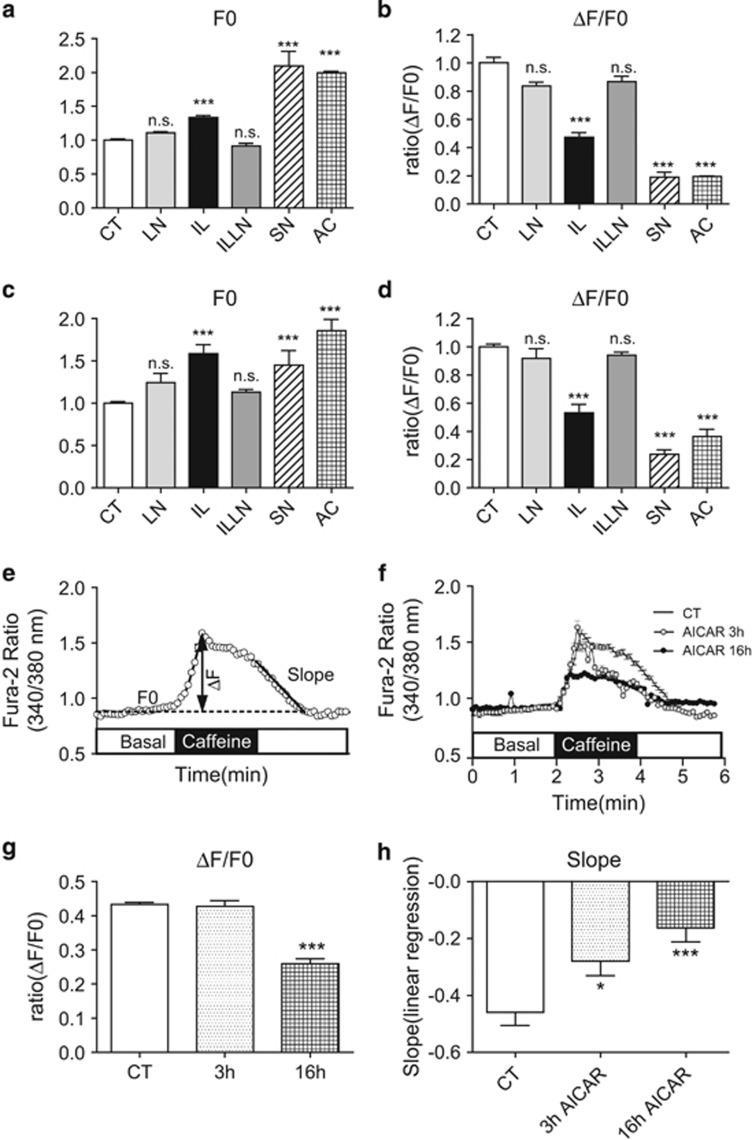
IL-1*β*, SNAP and AICAR alter *β-*cell calcium homeostasis, and direct activation of AMPK impairs SERCA2 activity. To assess cytosolic Ca^2+^ levels, Calcium 6 fluorescence and fura-2/AM fluorescence ratios were measured as described under Materials and Methods. For Calcium 6 measurements (**a**–**d**), INS-1 cells were pretreated with dimethyl sulfoxide (DMSO) (CT), 5 ng/ml IL-1*β* (IL) combined with or without 0.5 mM  l-NMMA (LN), 300 mM SNAP (SN) or 2 mM AICAR (AC) for 24 h. (**e**–**h**) For fura-2/AM assays, INS-1 cells were pretreated with DMSO (CT) or 2 mM AICAR for 3 or 16 h. Quantitative results of the basal cytosolic Ca^2+^ level (*F*0) and the relative ER Ca^2+^ extrusion described as a normalized ratio according to the formula Δ*F/F*0 performed in the presence (**a** and **b**) or absence (**c** and **d**) of 2.5 mM CaCl_2_ are shown. (**e**) Schematic illustrating the calculation of Δ*F/F*0 ratios from fura-2/AM imaging experiments. The Ca^2+^ clearance rate was used as an estimate of SERCA2 activity and was analyzed using linear regression to calculate the slope of the fura-2/AM ratio following withdrawal of caffeine. (**f**) Representative trace from untreated INS-1 cells (CT) and INS-1 cells treated with AICAR for 3 or 16 h. (**g**–**h**) Quantitative results of the Δ*F/F*0 ratio and slope from INS-1 cells untreated (CT) or treated with AICAR for 3 or 16 h. Indicated comparisons are significantly different (*P<0.01 and ***P<0.001)

To explore the functional effects of proinflammatory signaling on *β-*cell Ca^2+^ homeostasis, the FLIPR Calcium 6 Assay Kit (Molecular Devices, Sunnyvale, CA, USA) was used to measure basal cytosolic Ca^2+^ levels and Ca^2+^ mobilization from the ER in response to the indicated compounds (Figures 7a–d). Experiments were performed both in the presence (Figures 7a and b) and absence of extracellular Ca^2+^ (Figures 7c and d) and showed that IL-1*β*, SNAP and AICAR significantly increased basal cytosolic Ca^2+^ levels and decreased ER Ca^2+^ levels (as assessed by changes in the Δ*F/F*0 ratio following caffeine treatment). Notably, the effects of IL-1*β* were reversed by cotreatment with l-NMMA. In aggregate, these results demonstrate that proinflammatory NO-mediated signaling as well as AMPK activation alter *β*-cell Ca^2+^ compartmentalization, resulting in decreased ER Ca^2+^ levels and a reciprocal increase in basal cytosolic Ca^2+^.

Next, INS-1 cells were incubated with the Ca^2+^ dye fura-2/AM. Basal Ca^2+^ levels within the cytosolic compartment and changes in Ca^2+^ transit following ER Ca^2+^ depletion with caffeine were analyzed according to the schematic indicated in Figure 7e. To provide an additional estimate of SERCA pump activity, the slope of the change in the fura-2/AM ratio following withdrawal of caffeine and closure of RyR was calculated using linear regression (Figure 7e). INS-1 cells were then treated with AICAR for 0, 3 and 16 h. Whereas 3 h of AICAR treatment did not alter the Δ*F/F*0 ratio (Figure 7g), AICAR treatment for 16 h significantly reduced the Δ*F/F*0 ratio. Interestingly, both short-term (3 h) and long-term (16 h) AICAR led to decreased Ca^2+^ reuptake following removal of caffeine. Taken together, these results were consistent with observed changes in SERCA2b expression and suggest that AMPK activation impairs SERCA activity (Figure 7d), whereas chronic AMPK treatment leads to a reduction in ER calcium storage.

### Inhibition of iNOS and AMPK protect INS-1 cells from IL-1*β*-induced apoptosis

**Figure 8:**
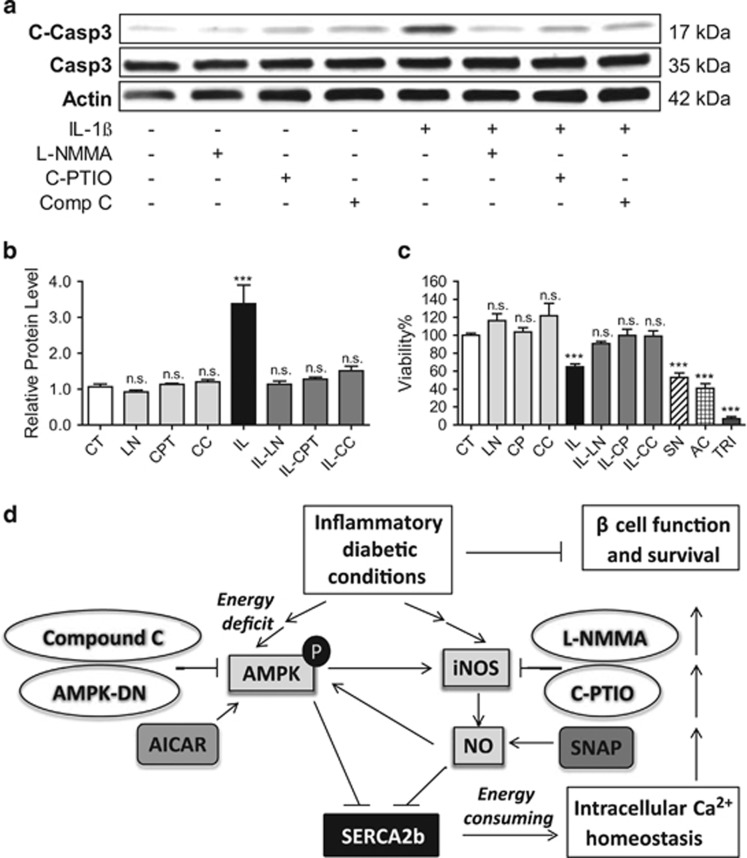
Inhibition of iNOS and AMPK protect INS-1 cells from IL-1*β*-induced apoptosis. INS-1 cells were treated with dimethyl sulfoxide (DMSO) (CT) or 5 ng/ml IL-1*β* (IL) combined with or without 0.5 mM of the NOS inhibitor l-NMMA (LN) or 100 *μ*M of the NO scavenger C-PTIO (CPT) for 24 h (**a**–**c**). Total protein was isolated, and immunoblot was performed using antibodies against cleaved caspase-3, total caspase-3 and actin. (**b**) Ratios of the relative expression of cleaved caspase-3 to total caspase-3 are shown graphically. (**c**) Cell viability assays were performed as described in the Materials and Methods section; 10% Triton treatment for 10 min was used as positive control for cell death. Indicated comparisons are significantly different (***P<0.001). (**d**) Our overall model suggests that under proinflammatory and diabetic conditions, activation of NO-mediated signaling in the pancreatic *β-*cell leads to an alteration in cellular energy status and activation of the master cellular energy sensor AMPK. The convergence of NO and AMPK signaling leads to a restraint of SERCA2b activity and altered SERCA2b protein stability, and the consequence of reduced SERCA2b expression and activity is dysregulation of intracellular and ER Ca^2+^ homeostasis that ultimately leads to a loss of *β-*cell function and survival. Whereas NO may initiate the activation of AMPK, our data also suggest that AMPK amplifies the inflammatory response of the pancreatic *β*-cell

Finally, to determine whether the observed rescue of SERCA2 expression also influenced cell survival, INS-1 cells were treated with l-NMMA, C-PTIO or CC alone or combined with IL-1*β*, and the ratio of cleaved caspase-3 to total caspase-3 expression was measured by immunoblot. Cleaved caspase-3 expression was significantly increased with IL-1*β* treatment, but reduced to control levels in the presence of l-NMMA, C-PTIO and CC (Figures 8a and b). Next, the CellTiter-Glo Luminescent Cell Viability Assay (Promega, Madison, WI, USA) was used in INS-1 cells treated with the same combination of compounds above as well as SNAP and AICAR alone. Viability was similarly reduced with IL-1*β*, SNAP and AICAR, whereas l-NMMA, C-PTIO and CC were able to rescue IL-1*β*-mediated cell death (Figure 8c), indicating that loss of *β*-cell survival under proinflammatory conditions occurs through NO- and AMPK-dependent pathways and is closely correlated with changes in SERCA2b expression and activity.

## Discussion

We and others have demonstrated diminished *β*-cell SERCA2b levels in human and rodent models of type 1 and type 2 diabetes,^1, 20, 21^ with alterations in SERCA2b expression leading to impaired Ca^2+^ homeostasis and insulin secretion, activation of ER stress signaling pathways and altered *β-*cell survival.^3, 5, 7, 8^ Ilham and co-workers^22^ previously descrbed a role for proinflammatory cytokines and induction of the NF-*κ*B-dependent gene, *iNOS*, in the loss of *β*-cell SERCA2b mRNA and protein expression. However, whether NO-mediated changes in SERCA2b expression and/or activity are primarily due to a transcriptional or posttranslational mechanism remains unexplored. Moreover, the downstream pathways that synergize with NO to alter endoplasmic reticulum Ca^2+^ in the *β-*cell have not been fully defined.

To address this, we first established the half-life of SERCA2 protein and mRNA under basal and proinflammatory conditions. Data from both INS-1 cells and rat islets demonstrate that treatment with the single cytokine IL-1*β* led to decreased SERCA2 protein stability without significantly altering mRNA half-life. Interestingly, treatment with the iNOS inhibitor, l-NMMA, was capable of rescuing SERCA2 protein under inflammatory conditions, but was unable to restore mRNA levels, suggesting divergent regulation of mRNA and protein expression under diabetic and inflammatory conditions. Indeed, our previous work is consistent with this notion and has shown that reductions in SERCA2b mRNA in models of diabetes may arise instead from the loss of key transcriptional regulators such Pdx-1 and PPAR-*γ*, rather than through decreased transcript stability.^5, 8^

Although temporally regulated and controlled increases in NO have important signaling effects in the pancreatic *β-*cell,^23^ chronically elevated NO generated under pathologic conditions has a central role in cytokine-induced *β*-cell death through a variety of effects, including decreased protein kinase B signaling, potentiation of c-Jun N-terminal kinase (JNK) activation and induction of irreversible DNA damage.^24, 25, 26^ However, a dominant effect of NO is impaired mitochondrial oxidation and altered ATP production, which occurs through inhibition of iron-sulfur enzymes such as aconitase.^27, 28^ These effects are quite potent in the pancreatic islet, where treatment with IL-1*β* is associated with at least a fourfold reduction in ATP levels.^29^

AMPK is a multisubstrate, heterotrimeric serine/threonine kinase with a role in a variety of cellular pathways. AMPK is activated directly through phosphorylation by one of three well-described upstream kinases, LKB1, CaMKK or TAK1, whereas a second major pathway of activation involves allosteric modulation by AMP and ADP. Thus, AMPK activity increases in response to any process that decreases ATP levels, including NO-induced stress, hypoxia and glucose deprivation.^30^ Interestingly, AMPK has also been shown to regulate ion transport as well as cellular ATPase activity. In this regard, Alzamora *et al.*^18^ demonstrated direct phosphorylation of the kidney vacuolar H^+^-ATPase by AMPK, and this modification led to changes in cellular localization and decreased activity of the protein pump. Similarly, hypoxia in alveolar cells leads to AMPK-triggered inactivation and endocytosis of the basolateral Na^+^,K^+^-ATPase, with resulting impairments in lung fluid clearance.^16^

With these relationships in mind, we investigated a novel link between SERCA2b and AMPK signaling, hypothesizing that restraint of SERCA activity may be one way that AMPK alters *β*-cell energy expenditure under inflammatory conditions. ATPases are energetically expensive to maintain and SERCA pumps alone have been estimated to consume upwards of 7–25% of cellular ATP under normal conditions.^10, 11, 12^ In support of our hypothesis, a recent interactome study involving large-scale affinity purification-mass spectrometry of the AMPK*α* subunit was performed in INS-1 *β*-cells and identified SERCA2 as a potential AMPK*α* binding partner.^19^ Indeed, our results show that chronic AMPK activation decreases SERCA2 protein expression in INS-1 cells, rat islets and human islets through a mechanism involving a reduction in protein half-life. Moreover, pharmacologic as well as genetic inhibition of AMPK was capable of rescuing cytokine-induced loss of SERCA2 expression and cytokine-induced cell death. We explored a functional effect of this relationship using intracellular Ca^2+^ imaging and demonstrate that both short- and long-term AMPK activation alters SERCA activity levels, leading to reductions in ER Ca^2+^ with a reciprocal increase in cytosolic Ca^2+^.

Although beneficial metabolic effects of AMPK have been well described in peripheral tissues including the liver and skeletal muscle,^30^ the effects of AMPK activation in the *β*-cell remain somewhat controversial. Divergent effects of AMPK appear to be dependent on both the level of activation as well as the chronicity of activation in experimental models. In the short-term, AMPK stimulates insulin secretion and may promote recovery from stress.^31^ In contrast, chronic or long-term activation, similar to the paradigm applied in our study, has been linked to impairments in insulin secretion as well as altered *β-*cell survival.^31, 32, 33^ Indeed, a proapoptotic role for AMPK has been described in other cell types and is linked with a variety of mechanisms including cell cycle arrest, activation of p53 and JNK signaling.^34^ Specifically in the *β-*cell and consistent with our findings, previous studies suggest that pharmacologic activation of AMPK with AICAR or virally induced constitutive activation of AMPK resulted in islet and *β-*cell death, whereas a dominant-negative form of AMPK (AMPK-DN) was capable of protecting against cytokine-induced apoptosis.^14, 35^ Similarly, constitutive activation of AMPK within transplanted islets decreased insulin secretion and *β-*cell survival in STZ-treated diabetic mice.^36^

Our study raises a number of interesting questions for future investigation. Although we suggest an initiating role for ATP depletion in our model, there are likely other pathways that lead to AMPK activation under inflammatory conditions. In fact, a study by Gordon *et al.*^33^ showed that NO modulates AMPK activity in the *β*-cell through an IRE-1-dependent mechanism as part of the unfolded protein response.^33^ It is also tempting to speculate that increased cytosolic Ca^2+^ resulting from initial pertubations in SERCA activity may augment AMPK activation through a CaMKK-dependent pathway, although this remains to be tested. Similarly, an interesting relationship uncovered by our data is that pharmacologic inhibition of AMPK with CC and treatment with the AMPK-DN construct attenuated IL-1*β*-induced iNOS gene and protein expression. This result was further confirmed by measuring nitrite concentrations in the culture media. Similar to our results, Santos and co-workers^31, 37^ have shown that knockdown of AMPK in MIN-6 *β-*cells blocked cytokine- or lipopolysaccharide-induced expression of iNOS. These data and ours suggest that AMPK is not only activated by NO signaling but may also be responsible for amplifying the inflammatory response. AICAR has previously been shown to activate NF-*κ*B in a neuroblastoma cell line through degradation of I*κ*B*α*.^34^ We investigated this possibility in INS-1 cells, and similarly found that AICAR treatment led to a dose-dependent reduction in I*κ*B*α* expression in the pancreatic *β*-cell.

In aggregate, these data provide evidence for a novel pathway that links NO and AMPK signaling with altered *β*-cell calcium homeostasis and provide additional insight into the regulation of *β*-cell survival under inflammatory conditions that typify both type 1 and type 2 diabetes.

## Materials and Methods

### Materials

CHX, actinomycin D and CC were purchased from Sigma-Aldrich (St Louis, MO, USA), whereas AICAR was from Toronto Research Chemicals (Toronto, ON, Canada). Recombinant mouse and human IL-1*β*, TNF-*α* and interferon-*γ* (IFN-*γ*) were purchased from Life Technology (Carlsbad, CA, USA), whereas caffeine and l-NMMA were from Santa Cruz Biotechnology (Santa Cruz, CA, USA). C-PTIO and SNAP were obtained from Cayman Chemical (Ann Arbor, MI, USA).

### Animals, islet isolation and human islets preparation

Male wistar rats (250–300 g) were purchased from Harlan Lab (Indianapolis, IN, USA), and maintained under protocols approved by the Indiana University Institutional Animal Care and Use Committee, the US Department of Agriculture's Animal Welfare Act (9 CFR Parts 1, 2 and 3) and the Guide for the Care and Use of Laboratory Animals.^38^ Rat pancreatic islets were isolated by collagenase digestion as described previously,^39^ hand-picked and allowed to recover overnight in RPMI-1640 medium supplemented with 10% fetal bovine serum, 100 U/ml penicillin and 100 *μ*g/ml streptomycin. Human cadaveric islets isolated from non-diabetic donors were obtained from the Integrated Islet Distribution Program or the National Disease Research Interchange. Upon receipt, human islets were hand-picked and allowed to recover overnight in DMEM medium containing 5.5 mM glucose, 10% fetal bovine serum, 100 U/ml penicillin and 100 *μ*g/ml streptomycin.

### Cell culture and *in vitro* islet treatment

INS-1 832/13 rat insulinoma cells were cultured as described previously.^7^ An AMPK-DN recombinant adenovirus expressing an HA-tagged human *α*1-subunit with a D159A mutation in the ATP-binding domain was purchased from Eton Biosciences (San Diego, CA, USA). An adenovirus encoding firefly luciferase under the control of the cytomegalovirus promoter was used as a control. For loss of function studies, INS-1 832/13 cells were transduced with 10^7^ PFU/ml of control or AMPK-DN adenovirus when cells reached 60–70% confluency. Rat, mouse or human islets were transduced on the day of isolation or receipt using 5 × 10^7^ PFU/ml of control or AMPK-DN adenovirus. After overnight incubation, the culture media was replaced with fresh media followed by an additional 24–32 h of culture. To simulate the inflammatory milieu of diabetes, cells and rat islets were treated in RPMI media containing 5 ng/ml of mouse IL-1*β* for indicated times. For human islet studies, DMEM media containing 5 ng/ml of mouse or human IL-1*β*, 10 ng/ml TNF-*α* and 100 ng/ml IFN-*γ* was used. Time-course experiments were performed to determine the half-life of SERCA2b protein or mRNA using 10 *μ*M CHX or 1 *μ*g/ml actinomycin D, respectively. The CellTiter-Glo Luminescent Cell Viability Assay Kit was used according to the manufacturer's instructions. NO generation was measured using the Promega Griess Reagent System (Madison, WI, USA).

### Quantitative real-time PCR and immunoblot analysis

Cultured INS-1 832/13 cells or isolated rat islets and human islets were washed with PBS and quantitative RT-PCR was performed using previously published methods and primer sequences.^5^ Immunoblot analysis was performed as described previously using the following primary antibodies: anti-SERCA2 goat antibody (1:1000; Santa Cruz Biotechnology); anti-phospho-AMPK*α* Th172 rabbit antibody (1 : 1000; Cell Signaling, Beverly, MA, USA); anti-total AMPK*α* rabbit antibody (1 : 1000; Cell Signaling); anti-total acetyl-CoA carboxylase (ACC) antibody (1 : 1000; Cell Signaling); anti-phospho-ACC (1:1000; Cell Signaling); anti-total caspase-3 rabbit antibody, which detected both cleaved and total caspase-3 (1 : 1000; Cell Signaling); anti-iNOS rabbit antibody (1 : 1000; Millipore, Billerica, MA, USA); and anti-actin mouse antibody (1 : 10 000; MP Biomedicals, Santa Ana, CA, USA).

### Cytoplasmic calcium (Ca^2+^) imaging

Intracellular cytosolic Ca^2+^ was measured using the FLIPR Calcium 6 Assay Kit (Molecular Devices) according to the manufacturer's instructions. In brief, INS-1 832/13 cells were plated at a density of 5000 cells per well in black wall/clear bottom 96-multiwell plates from Costar (Tewksbury, MA, USA) and cultured for 2 days before treatment. After ~22 h of treatment, Calcium 6 reagent was added directly to cells, and cells were incubated for an additional 2 h at 37 °C and 5% CO_2._ Data acquisition on the FlexStation 3 system (Molecular Devices) was performed at room temperature using a 1.52- s reading interval throughout the experiments. To empty ER Ca^2+^ stores, caffeine dissolved in Hank's balanced salt solution (HBSS) was injected into each well to achieve a final concentration of 10 mM, and fluorescence was recorded for an additional 120 s. To image under Ca^2+^ free conditions, following 2 h of incubation with the Calcium 6 reagent, media were substituted with HBSS without Ca^2+^ before data acquisition.

To provide an additional measure of intracellular Ca^2+^ mobilization, the ratiometric calcium indicator fura-2/AM from Life Technologies as described previously^40^ was applied. In brief, INS-1 832/13 cells were seeded in glass bottom 50 mm plates for 48 h and then treated as indicated. Before imaging, INS-1 cells were incubated at 37 °C and 5% CO_2_ in 4 *μ*M fura-2/AM and 0.02% pluronic F127 (Life Technologies) for 1 h, and then washed and incubated with HBSS (Life Technologies) supplemented with 0.1% BSA and 2.5 mM CaCl_2_. A small-volume chamber from Warner Instruments (Hamden, CT, USA) was mounted on the microscope stage; INS-1 cells were perfused with a gradient pump (Bio-Rad, Hercules, CA, USA) and maintained at 37 °C and 5% CO_2_. To activate ryanodine receptors and reversibly empty ER Ca^2+^ stores, 10 mM caffeine in basal HBSS was used for 2 min. Fura-2/AM fluorescence was measured with excitation at 340 and 380 nm and emission at 510 nm, and images were captured using a Zeiss Z1 microscope (Carl Zeiss Microscopy, Oberkochen, Germany) with a x10 objective. Results were analyzed with Zen Blue software (Zeiss, Oberkochen, Germany). For both Calcium 6 and fura-2/AM experiments, basal Ca^2+^ (*F*0) measurements and Max-Min Ca^2+^ measurements (Δ*F*) were assessed as described previously.^8^

### Statistical analysis

Results are displayed as the means±S.E.M., and at least three sets of replicates were performed for each experiment. GraphPad Prism statistics software (GraphPad Software, La Jolla, CA, USA) was used to draw one-phase decay curves for protein and mRNA half-life determination and for linear regression of the Ca^2+^ reuptake response following caffeine withdrawal. Differences between groups were examined for significance using either a two-tailed Student's *t*-test or one-way ANOVA followed by the Tukey–Kramer posttest. A *P-*value <0.05 was taken to indicate the presence of a significant difference between groups.

## Abbreviations

RyR: ryanodine receptor
ER: endoplasmic reticulum
SERCA: sarcoendoplasmic reticulum calcium ATPase
NO: nitric oxide
SNAP: *S*-nitroso-*N*-acetyl-d,l-penicillamine
AMPK: AMP-activated protein kinase
C-PTIO: carboxylated derivative of 2-phenyl-4,4,5,5-tetramethylimidazoline-1-oxyl-3-oxide
l-NMMA: *NG*-monomethyl l-arginine
AICAR: 5-aminoimidazole-4-carboxamide ribonucleotide
ACC: acetyl-CoA carboxylase
iNOS: inducible nitric oxide synthase
HBSS: Hank's balanced salt solution
IL-1*β*: interleukin-1*β*
TNF-*α*: tumor necrosis factor-*α*
IFN-*γ*: interferon-*γ*
CC: compound C
